# Supplementary figures and images for: Transforming a fragile protein helix into an ultrastable scaffold via a hierarchical AI and chemistry framework
Source: eLife. 2026 Apr 2;15:RP109753. doi: 10.7554/eLife.109753 (PMC13046381; doi:10.7554/eLife.109753)

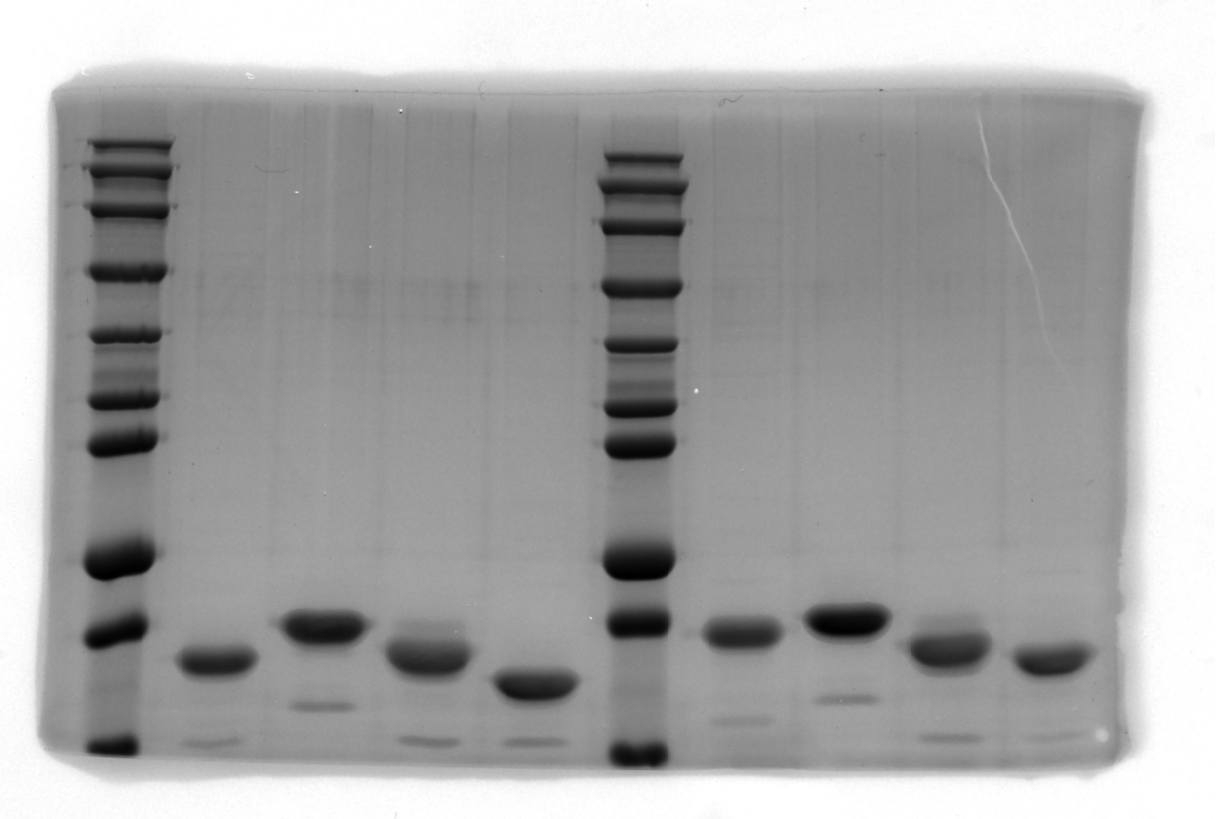

Supplement: Figure 2—figure supplement 1—source data 1. [file elife-109753-fig2-figsupp1-data1.png]

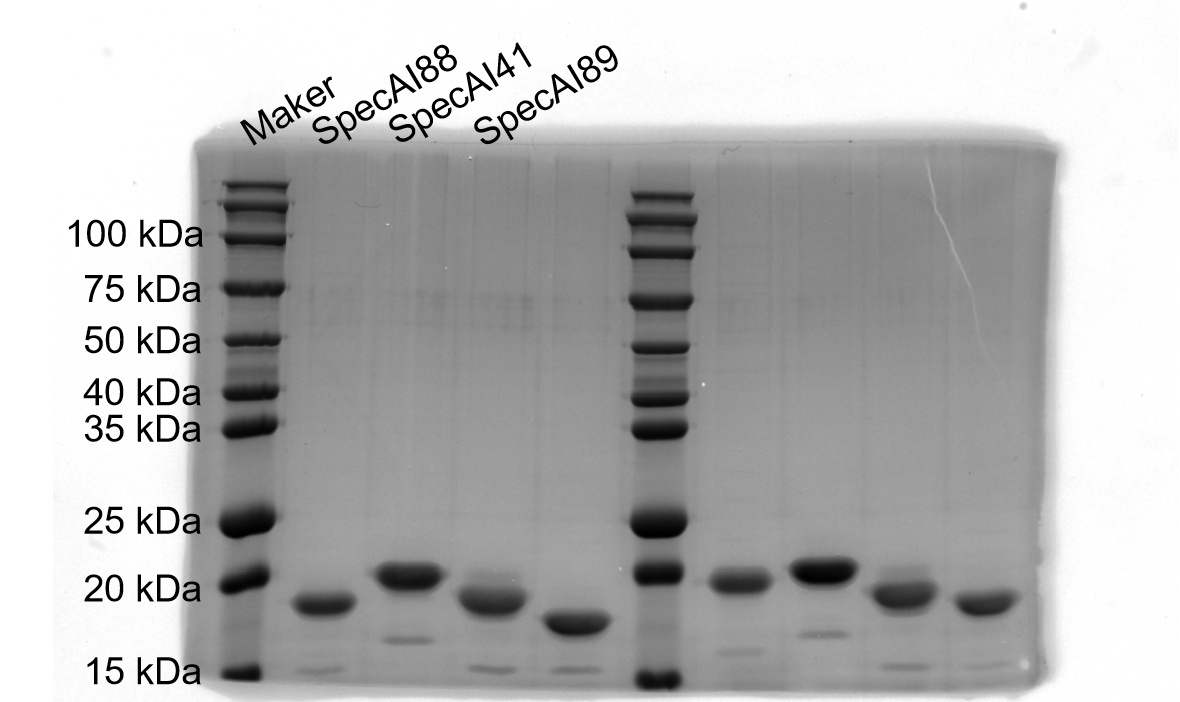

Supplement: Figure 2—figure supplement 1—source data 2. [file elife-109753-fig2-figsupp1-data2.png]

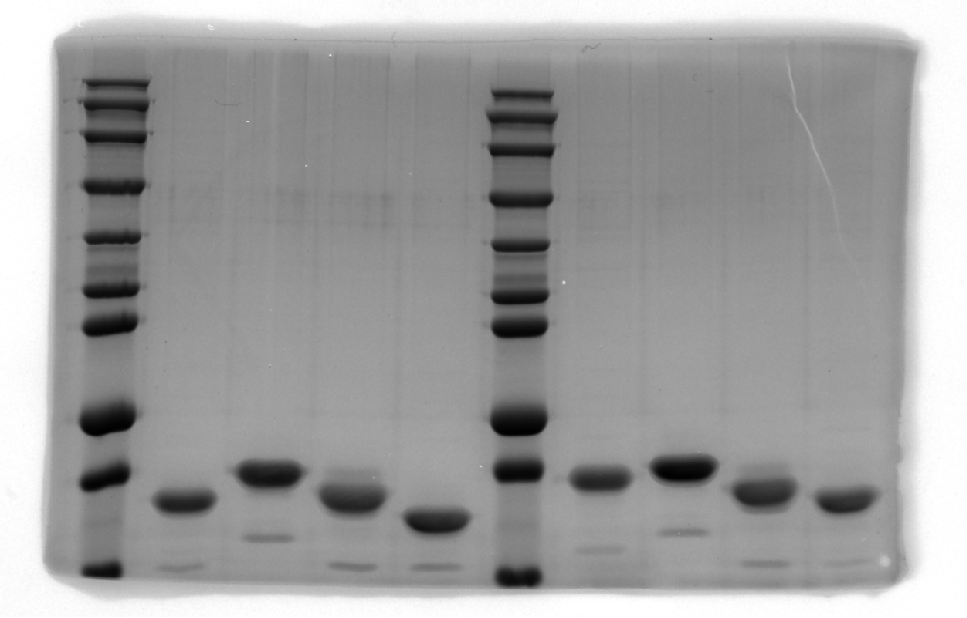

Supplement: Figure 4—figure supplement 1—source data 1. [file elife-109753-fig4-figsupp1-data1.png]

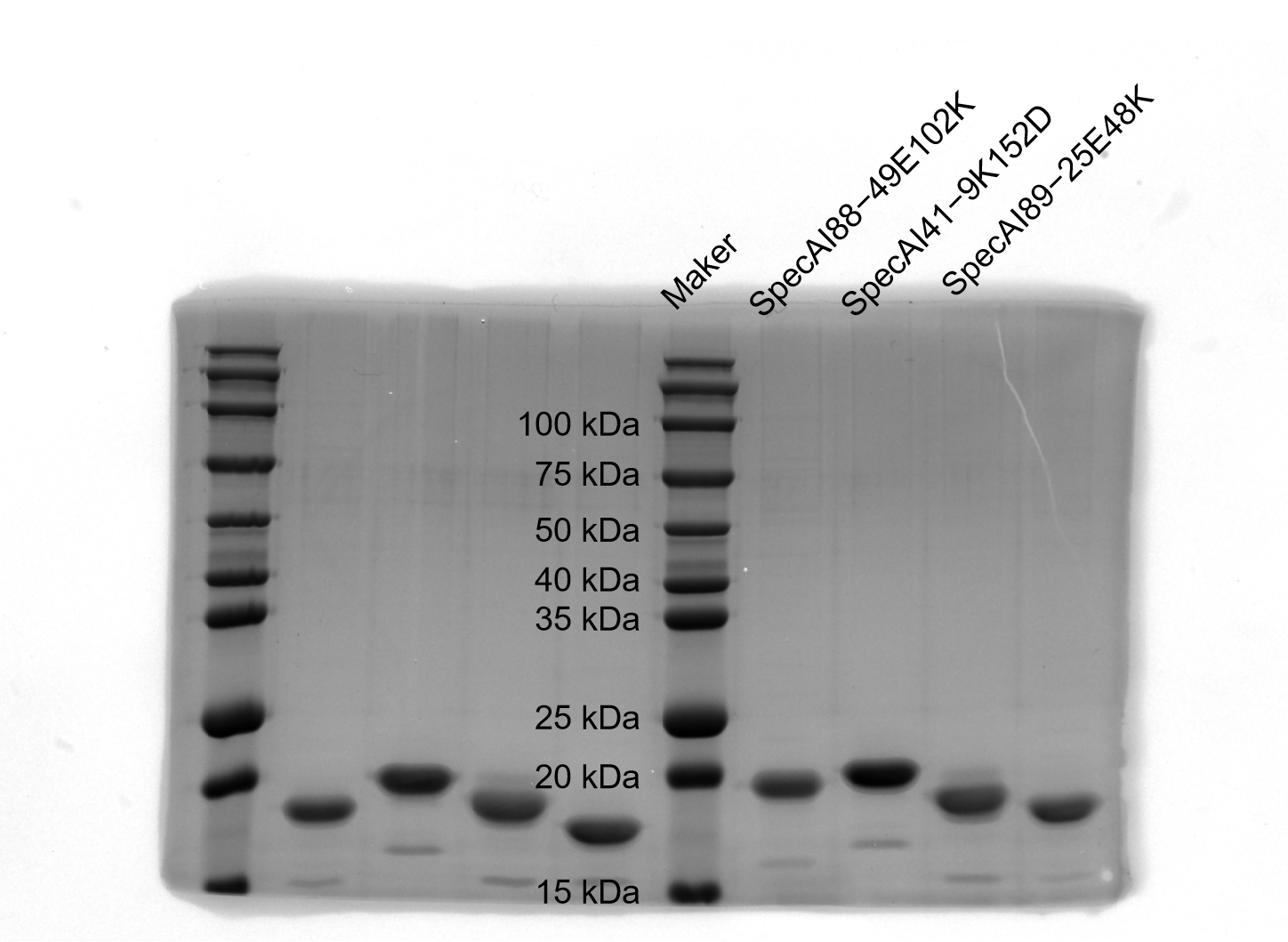

Supplement: Figure 4—figure supplement 1—source data 2. [file elife-109753-fig4-figsupp1-data2.png]

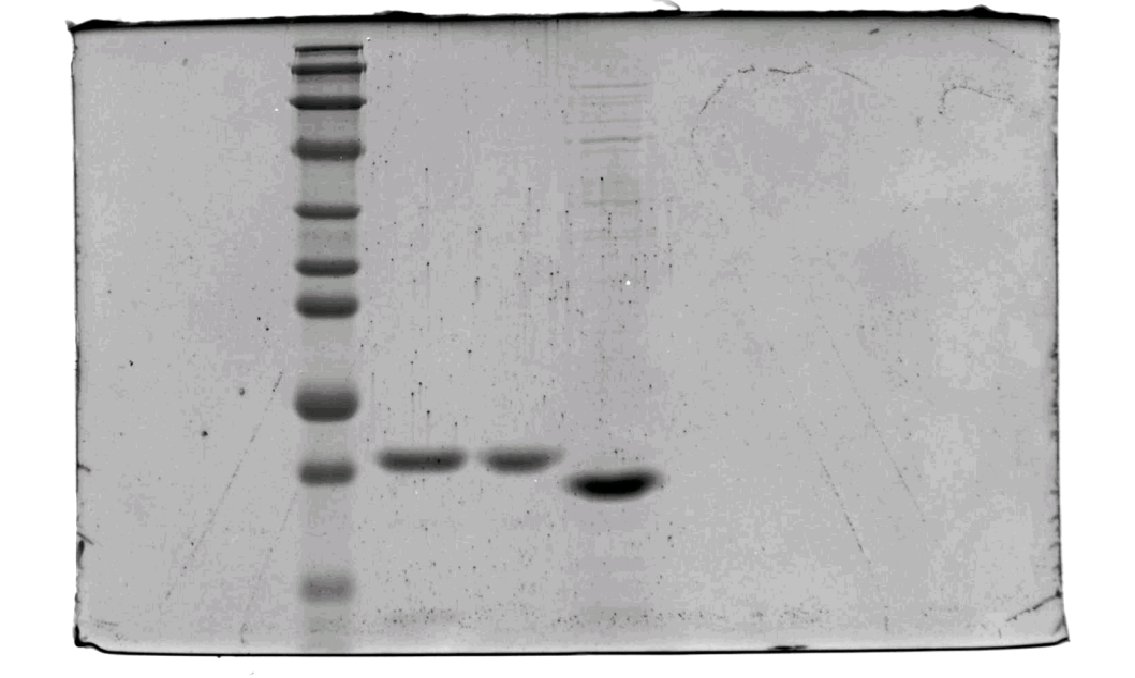

Supplement: Figure 4—figure supplement 2—source data 1. [file elife-109753-fig4-figsupp2-data1.png]

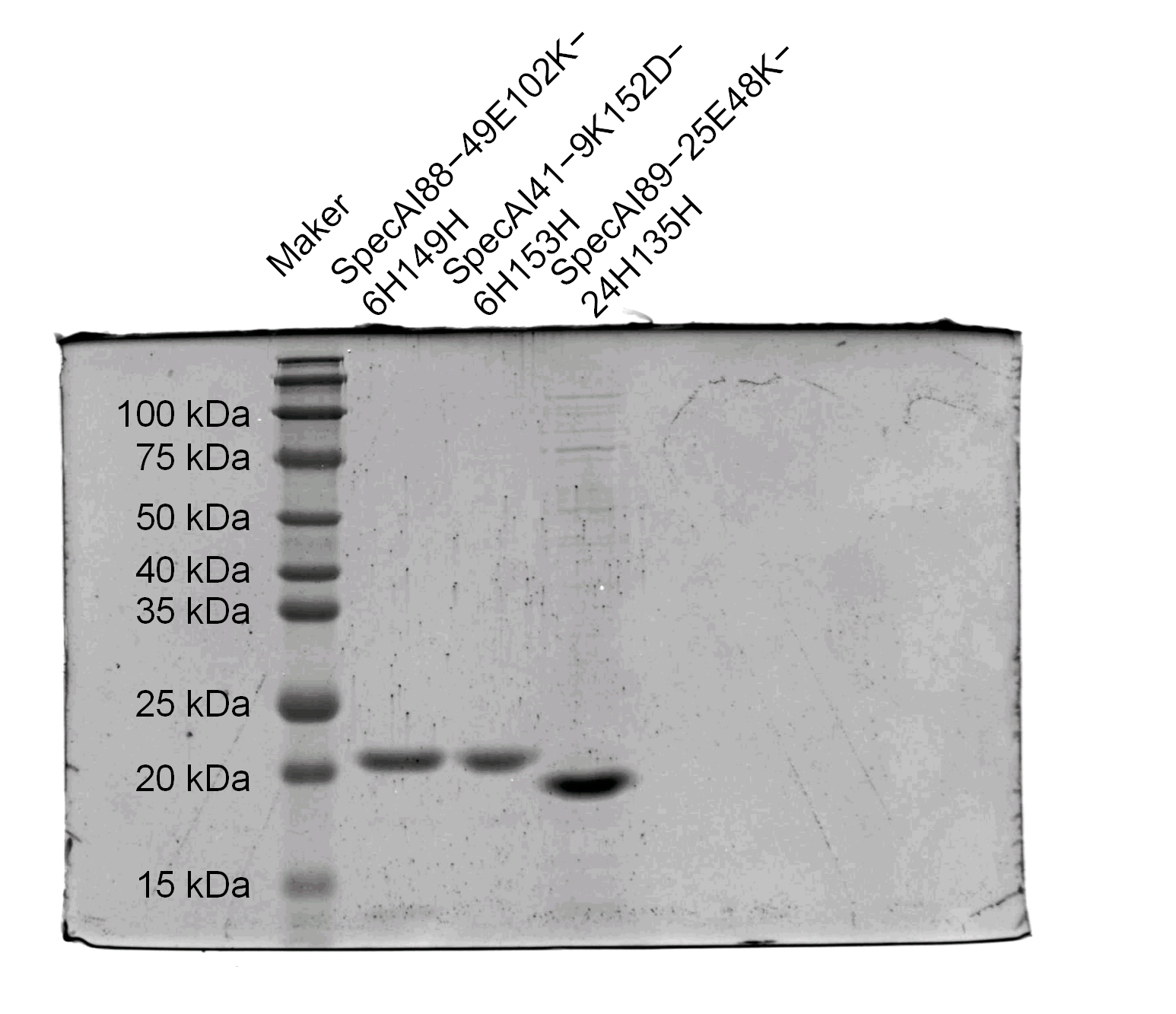

Supplement: Figure 4—figure supplement 2—source data 2. [file elife-109753-fig4-figsupp2-data2.png]
