# Supplementary material for: Transforming a fragile protein helix into an ultrastable scaffold via a hierarchical AI and chemistry framework
Source: eLife. 2026 Apr 2;15:RP109753. doi: 10.7554/eLife.109753 (PMC13046381; doi:10.7554/eLife.109753)
Supplement: Supplementary file 1. [file elife-109753-supp1.docx]

**Supplementary file 1. Protein sequences for AFM-SMFS measurement.**

Protein sequence of **GL**-**His6**-**GB1**-**Xmod**-**Doc** (Molecular Weight, MW: 33.4 *k*Da):

**GLHHHHHH**GS**YKLILNGKTLKGETTTEAVDAATAEKVFKQYANDNGVDGEWTYDDATKTFTVTE**RSGG**NTVTSAVKTQYVEIESVDGFYFNTEDKFDTAQIKKAVLHTVYNEGYTGDDGVAVVLREYESEPVDITAELTFGDATPANTYKAVENKFDYEIPVYYNNATLKDAEGNDATVTVYIGLKGDTDLNNIVDGRDATATLTYYAATSTDGKDATTVALSPSTLVGGNPESVYDDFSAFLSDVKVDAGKELTRFAKKAERLIDGRDASSILTFYTKSSVDQYKDMAANEPNKLWDIVTGDA**RS

Protein sequence of **GL**-**His6**-**ELP20**-**Cys** (MW: 9.7 *k*Da):

M**GLHHHHHH**GS**VPGVGVPGVGVPGVGVPGVGVPGVGVPGAGVPGAGVPGGGVPGGGVPGEGVPGEGVPGVGVPGVGVPGVGVPGVGVPGAGVPGAGVPGGGVPGGGVPGVG**RS**C**

Protein sequence of **His6**-**Cys**-**ELP20**-**NGL** (MW: 9.8 *k*Da):

MG**HHHHHHC**GS**VPGVGVPGVGVPGVGVPGVGVPGVGVPGAGVPGAGVPGGGVPGGGVPGEGVPGEGVPGVGVPGVGVPGVGVPGVGVPGAGVPGAGVPGGGVPGGGVPGVG**RS**NGL**

Protein sequence of **Coh**-**GB1**-**SpecAI88**-**GB1**-**His6**-**NAL** (MW: 55.0 *k*Da):

**GTALTDRGMTYDLDPKDGSSAATKPVLEVTKKVFDTAADAAGQTVTVEFKVSGAEGKYATTGYHIYWDERLEVVATKTGAYAKKGAALEDSSLAKAENNGNGVFVASGADDDFGADGVMWTVELKVPADAKAGDVYPIDVAYQWDPSKGDLFTDNKDSAQGKLMQAYFFTQGIKSSSNPSTDEYLVKANATYADGYIAIKAGEP**GGGGS**YKLILNGKTLKGETTTEAVDAATAEKVFKQYANDNGVDGEWTYDDATKTFTVTE**GSGS**KEEEILNWVKEFKEFWEWLAKRTKELAEDKVEDTGDPEAAREKQFEELLKEQEEREPLWEKLKEKTEELKKLNHPLYKELVKLAEFAEEVWELFKELFDYFKERQKLKAEGAPQEEIDKAKAKEEAALQKLKDIAPEVEKRKEELEKVVEEILK**RSGGGSGT**YKLILNGKTLKGETTTEAVDAATAEKVFKQYANDNGVDGEWTYDDATKTFTVTE**GS**HHHHHH**GGGGS**NAL**

Protein sequence of **Coh**-**GB1**-**SpecAI41**-**GB1**-**His6**-**NAL** (MW: 54.2 *k*Da):

**GTALTDRGMTYDLDPKDGSSAATKPVLEVTKKVFDTAADAAGQTVTVEFKVSGAEGKYATTGYHIYWDERLEVVATKTGAYAKKGAALEDSSLAKAENNGNGVFVASGADDDFGADGVMWTVELKVPADAKAGDVYPIDVAYQWDPSKGDLFTDNKDSAQGKLMQAYFFTQGIKSSSNPSTDEYLVKANATYADGYIAIKAGEP**GGGGS**YKLILNGKTLKGETTTEAVDAATAEKVFKQYANDNGVDGEWTYDDATKTFTVTE**GSGS**EEEKIKQWVKDFKEYFDWLAERTAELAADVVEDTGDPEAAREAREEELEKEFEEKKPIREKLEKEAEKLKKLNHPLYKELVEYAEYAKEIWALFEALFEARAKLKELKAKGAPKAEIDAAQAKRDAALAELKAKAPEVEAKRAKVEKVVEKILK**RSGGGSGT**YKLILNGKTLKGETTTEAVDAATAEKVFKQYANDNGVDGEWTYDDATKTFTVTE**GS**HHHHHH**GGGGS**NAL**

Protein sequence of **Coh**-**GB1**-**SpecAI89**-**GB1**-**His6**-**NAL** (MW: 54.4 *k*Da):

**GTALTDRGMTYDLDPKDGSSAATKPVLEVTKKVFDTAADAAGQTVTVEFKVSGAEGKYATTGYHIYWDERLEVVATKTGAYAKKGAALEDSSLAKAENNGNGVFVASGADDDFGADGVMWTVELKVPADAKAGDVYPIDVAYQWDPSKGDLFTDNKDSAQGKLMQAYFFTQGIKSSSNPSTDEYLVKANATYADGYIAIKAGEP**GGGGS**YKLILNGKTLKGETTTEAVDAATAEKVFKQYANDNGVDGEWTYDDATKTFTVTE**GSGS**SEKEIQEWVKEFKDAFDKLVERTEEIAADKVEPTGDPEKARDAKEEELLKEFEEFKPKREKLLKKAEKLKELNHPLYEKLVELSEFFEEQWALFEKYFAAFKKLKALKAEGAPQEEIDKARAERDAALAEIQAKAPEVKAKKEELDKVVEEILK**RSGGGSGT**YKLILNGKTLKGETTTEAVDAATAEKVFKQYANDNGVDGEWTYDDATKTFTVTE**GS**HHHHHH**GGGGS**NAL**

**Protein sequences for CD and MS measurements.**

Protein sequence of **SpecAI88**-**His6** (MW: 19.9 *k*Da):

MGS**KEEEILNWVKEFKEFWEWLAKRTKELAEDKVEDTGDPEAAREKQFEELLKEQEEREPLWEKLKEKTEELKKLNHPLYKELVKLAEFAEEVWELFKELFDYFKERQKLKAEGAPQEEIDKAKAKEEAALQKLKDIAPEVEKRKEELEKVVEEILK**GT**HHHHHH**

Protein sequence of **SpecAI41**-**His6** (MW: 19.1 *k*Da):

MGS**EEEKIKQWVKDFKEYFDWLAERTAELAADVVEDTGDPEAAREAREEELEKEFEEKKPIREKLEKEAEKLKKLNHPLYKELVEYAEYAKEIWALFEALFEARAKLKELKAKGAPKAEIDAAQAKRDAALAELKAKAPEVEAKRAKVEKVVEKILK**GT**HHHHHH**

Protein sequence of **SpecAI89**-**His6** (MW: 19.3 *k*Da):

MGS**SEKEIQEWVKEFKDAFDKLVERTEEIAADKVEPTGDPEKARDAKEEELLKEFEEFKPKREKLLKKAEKLKELNHPLYEKLVELSEFFEEQWALFEKYFAAFKKLKALKAEGAPQEEIDKARAERDAALAEIQAKAPEVKAKKEELDKVVEEILK**GT**HHHHHH**
